# Modified nodal stage of esophageal cancer based on the evaluation of the hazard rate of the negative and positive lymph node

**DOI:** 10.1186/s12885-020-07664-w

**Published:** 2020-12-07

**Authors:** Jinling Zhang, Hongyan Li, Liangjian Zhou, Lianling Yu, Fengyuan Che, Xueyuan Heng

**Affiliations:** 1 Cancer Center of Linyi People’s Hospital, Shandong University, School of Medicine, Linyi, 276000 Shandong Province P. R. China; 2 Department of Central Laboratory, Linyi People’s hospital, Shandong University, School of medicine, Linyi, 276000 Shandong Province, P. R. China

**Keywords:** Esophageal carcinoma, SEER, Tumor metastasis, TNM

## Abstract

**Background:**

The study aimed to propose a modified N stage of esophageal cancer (EC) on the basis of the number of positive lymph node (PLN) and the number of negative lymph node (NLN) simultaneously.

**Method:**

Data from 13,491 patients with EC registered in the SEER database were reviewed. The parameters related to prognosis were investigated using a Cox proportional hazards regression model. A modified N stage was proposed based on the cut-off number of the re-adjusted ratio of the number of PLN (_number_PLN) to the number of NLN (_number_NLN), which were derived from the comparison of the hazard rate (HR) of _number_PLN and _number_NLN. The modified N stage was confirmed using the cross-validation method with the training and validation cohort, and it was also compared to the N stage from the American Joint Committee on Cancer (AJCC) staging system (7th edition) using Receiver Operating Characteristic (ROC) curve analysis.

**Results:**

The _number_PLN on prognosis was 1.042, while _number_NLN was 0.968. The modified N stage was defined as follows: N1 stage: the ratio range was from 0 to 0.21; N2 stage: more than 0.21, but no more than 0.48; N3 stage: more than 0.48. The log-rank test indicated that significant survival differences were confirmed among the N1, N2 and N3 sub-groups of patients in the training population. The difference of all the patients using the modified N stage method were more significant than AJCC N stage. The result of ROC analysis indicated that the modified N stage could represent the N stage of EC more accurately.

**Conclusion:**

The modified N stage based on the re-adjusted ratio of _number_PLN to _number_NLN can evaluate tumor stage more accurately than the traditional N stage.

## Background

Eesophageal cancer (EC) is a fatal disease with a poor prognosis [1]. Lymph node (LN) metastasis usually occurs in the beginning of diagnosis, and accurate evaluation of the tumor stage is a key step in determining post-operation treatment [2]. However, at present, the definition of N stage is controversial. The N stage has typically been defined by the American Joint Committee on Cancer (AJCC) as the number of positive lymph node (PLN),but a new N stage was proposed by the Japanese Society for Esophageal Diseases (JSED) [3, 4]. The JSED N stage is defined according to the site of PLN. The site of PLN has been demonstrated to play an important role in the prognosis of patients with EC, while the key role of number of PLN on the prognosis of patients with EC was repeatedly confirmed and widely received by researchers [5–7]. Furthermore, recent research revealed that the site of PLN was weaker than the number of PLN (_number_PLN) in the multiple-parameters analysis using a survival model of EC [8]. Nevertheless, neither of them considered the influence of the number of negative lymph node (NLN) on the prognosis of patients with EC.

Greenstein first proposed the impact of the number of NLN (_number_NLN) on the outcome of patients with EC [9]. He suggested that the higher the NLN resected in during surgery would be associated with better post-operative outcome for patients. Hsu confirmed the above identification again in EC [10]. In another study, _number_NLN was included in a scoring system for determining the prognosis of EC [11]. In other words, it was accepted that _number_NLN counted in the operation could increase the accuracy of identifying the N stage of AJCC. It was also inferred that _number_NLN could represent site information for tumor metastasis to some extent.

Because of the significant impact _number_PLN and NLN has on the prognosis of patients with EC, a modified N stage that consists of both PLN and NLN might provide a more accurate representation of the extent of tumor metastasis in the regional LN station. However, it might not be accurate to define the modified N stage using the ratio of _number_PLN and NLN directly.

This study investigates the feasibility of a modified N stage which is based on a combination analysis including the number of positive LN and negative LN in the meantime. The combined analysis refer to the result of the Cox proportional hazard regression model.

## Methods

### Data source

The Surveillance, Epidemiology, and End Results (SEER) program of the National Cancer Institute is a comprehensive source of population-based cancer information in the United States. The SEER database collects disease incidence, patient treatments, and survival data from population-based cancer registries covering around 28% of the country’s population. SEER data comes primarily from hospital medical records as well as records from outpatient surgical, pathology, and radiology centers. The routine data collected in SEER database includes detailed information on demographics, diagnosis, and tumor characteristics. The work team engaged in active follow-up on the cases included in the SEER.

### Inclusion criteria for patients

This study reviewed patient information collected from 2004 to 2011. Data was downloaded using the SEER*Stat software (8.3.5, The Surveillance Research Program of the Division of Cancer Control and Population Sciences, National Cancer Institute.). The inclusion criteria in this study are as follows: 1) All patients should have experienced radical lymphadenectomy; 2) The LN number collected during the operation was clear; 3) EC is one of the specific causes of patient death.

A wide range of patient information was obtained from the SEER database. More specifically, the following variables and covariates were collected for this study: age, gender, tumor size, tumor extension, regional nodes positive, regional nodes negative, race (white, black and other), primary site of tumor (cervical segment, chest segment, abdominal segment and cross-section?), grade classification of tumor (I, II,III, and IV), AJCC Group (I, II,III, and IV), radiation sequence with surgery (no radiation and/or cancer-directed surgery, radiation prior to surgery, radiation after surgery, radiation before and after surgery, surgery both before and after radiation, sequence unknown, but both were given), tumor metastasis to bone, tumor metastasis to brain, tumor metastasis to liver, tumor metastasis to lung, the survival time and the status of patients.

### Statistical analysis

The total population was divided into two groups using a random number table. One group was the training population, and the other was the validation population. The cross-validation method was used between the training population and the validation population.

Cox proportional regression model was used to build a prediction function for time event data. The prediction function including the HR of PLN and NLN provided the coefficient to calculate the re-adjusted number of PLN and NLN for proposing the modified N stage of EC. The cutoff number for the ratio of the PLN count to NLN count was investigated using the method of the minimum of *P* values. This was performed using the software X-tile (2.0, University of Chicago).

Differences in survival rates between subgroups categorized by N stage were analyzed using the Kaplan–Meier analysis and log-rank test. Receiver Operating Characteristic (ROC) Curve Analysis was used to investigate whether the modified N stage proposed by this study was more effective than the previous N stage definition.

All analyses were performed using IBM SPSS version 21.0(SPSS Inc. Chicago, Illinois, USA). Continuous variables were presented as the mean ± standard deviation (SD) or when the data exhibited a skewed distribution, as the median and interquartile range (IQR). *P* values of 0.05(two-tailed) were established as the threshold for statistical significance.

## Results

### Baseline characteristics and outcomes

The data for around 100,000 Patients with EC were reviewed through SEER statistical software, but only the medical records of 13,491 patients were collected under the inclusion criteria**.** The 13,491 patients with EC were classified into two groups according to the random number method. The two groups were the training population (*n* = 6698) and the validation population (*n* = 6793).

The mean age of the total population was 66.70 ± 11.12 years, and 10,776(79.9%) of the patients were male. The average tumor size was 366.58 ± 179.92. The proportions of white, black, and other races were 84.9, 10.1, 4.7%, respectively. Follow-up data revealed that 5327(39.5%) of patients survived, and 8164(60.5%) of patients had died. Average survival time was 11.31 ± 11.35 months.

In the comparison of the results from the training population with that from the validation population, no significant differences were observed according to sex, age, tumor site, tumor size, the organ metastasis, _number_PLN and NLN (Table 1).

**Table 1:**
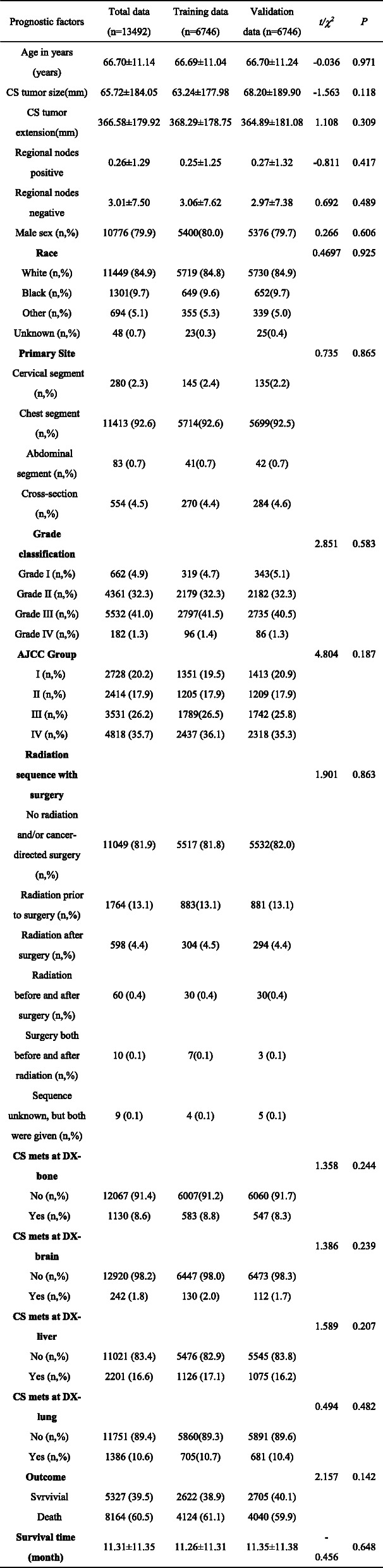
Baseline Characteristics of Subjects in the Training and Validation Data

### The parameters identified in the results of cox proportional Hazard regression analysis

The univariate analysis revealed that sex, race, age, tumor site, tumor size, tumor length, pathological grade of tumor, AJCC stage, the post-operation treatment, the distance of metastasis, and _number_NLN were all independent prognostic factors. The result of the multivariate analysis demonstrated that _number_PLN was also an independent prognostic factor in addition to the above parameters (Table 2).

**Table 2:**
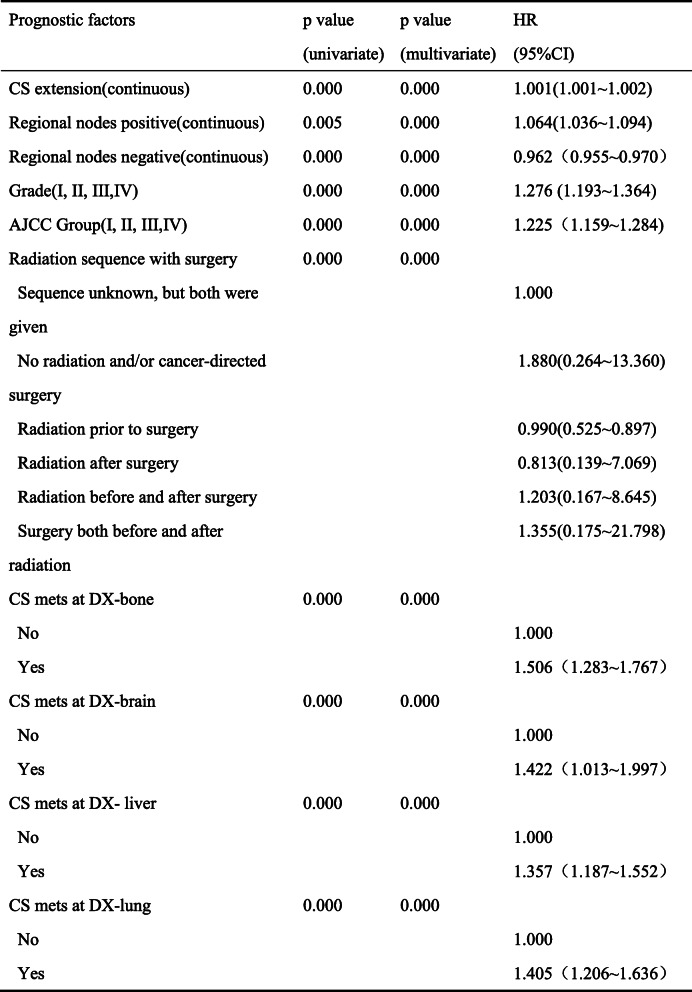
Univariate and multivariate for overall survival: Cox proportional hazard regression model

### The modified N stage proposed in this study

According to the results of the Cox proportional hazard model, the HR of PLN was 1.064, and the HR of NLN was 0.962. The distance between the HR of PLN and the statistical standard point (which was 1) was 0.064 (∆HP_positive_), and the distance between the HR of NLN and the statistical point was 0.038 (∆HP_negative_). The ratio of ∆HP_positive_ to ∆HP_negative_ (N ratio) was used as a coefficient to produce the re-adjusted number of PLN and NLN.

The analysis result of the minimum *P* value method indicated that the following ranges for the modified N stage were an appropriate solution:

- forN0 stage, the re-adjusted N ratio = 0;
- for N1stage, the re-adjusted N ratio = (0–0.08];
- for N2 stage, the range of rate was (0.08–0.63];
- for N3,the re-adjusted N ratio (0.63,+∞]

In order to calculate the ratio of the re-adjusted number of PLN to NLN in special situations, the authors settled on the following two definitions of the ratio:

1. When _number_PLN and NLN were both 0, the ratio of the readjusted number of PLN to NLN (re-adjusted N ratio) was defined as 0;
2. When one of _number_PLN and _number_NLN is 0, 0 was defined as 0.0001.

### The feasibility and superiority of the modified N stage

**Fig. 1:**
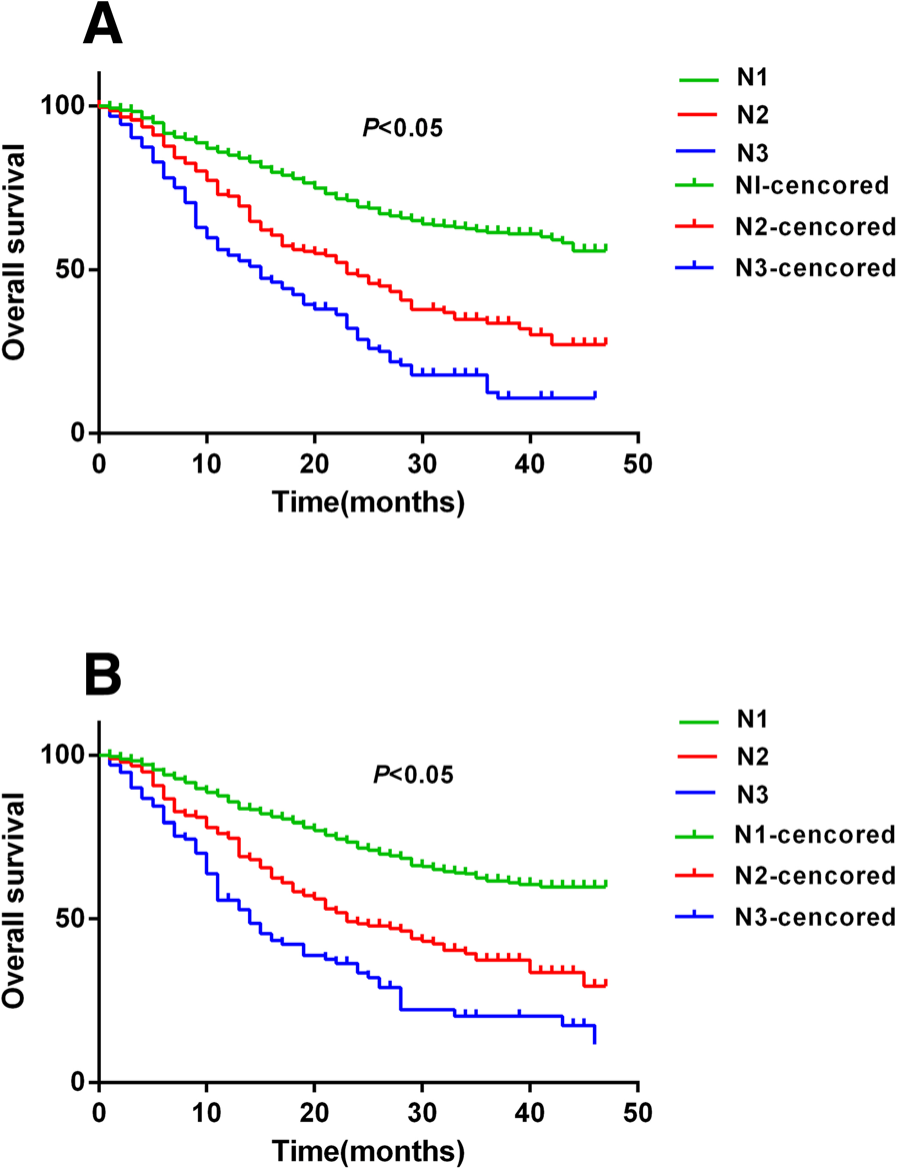
The comparison of overall survival between training group and validation group. **a*** The survival difference among N1, N2 and N3 sub group in training data were significant(P < 0.05).* **b*** The survival difference among N1, N2 and N3 sub group in validation data were significant(P < 0.05)*

A cross-validation study was performed on the modified N stage. The modified N stage was developed from the training population and validated using the validation population. The log-rank test indicated that significant survival differences were confirmed among the N1, N2 and N3 sub-groups of patients in the training population, and the survival difference could be replicated in the validation population using the Kaplan-Meier analysis (*P* < 0.05, Fig. 1).

**Fig. 2:**
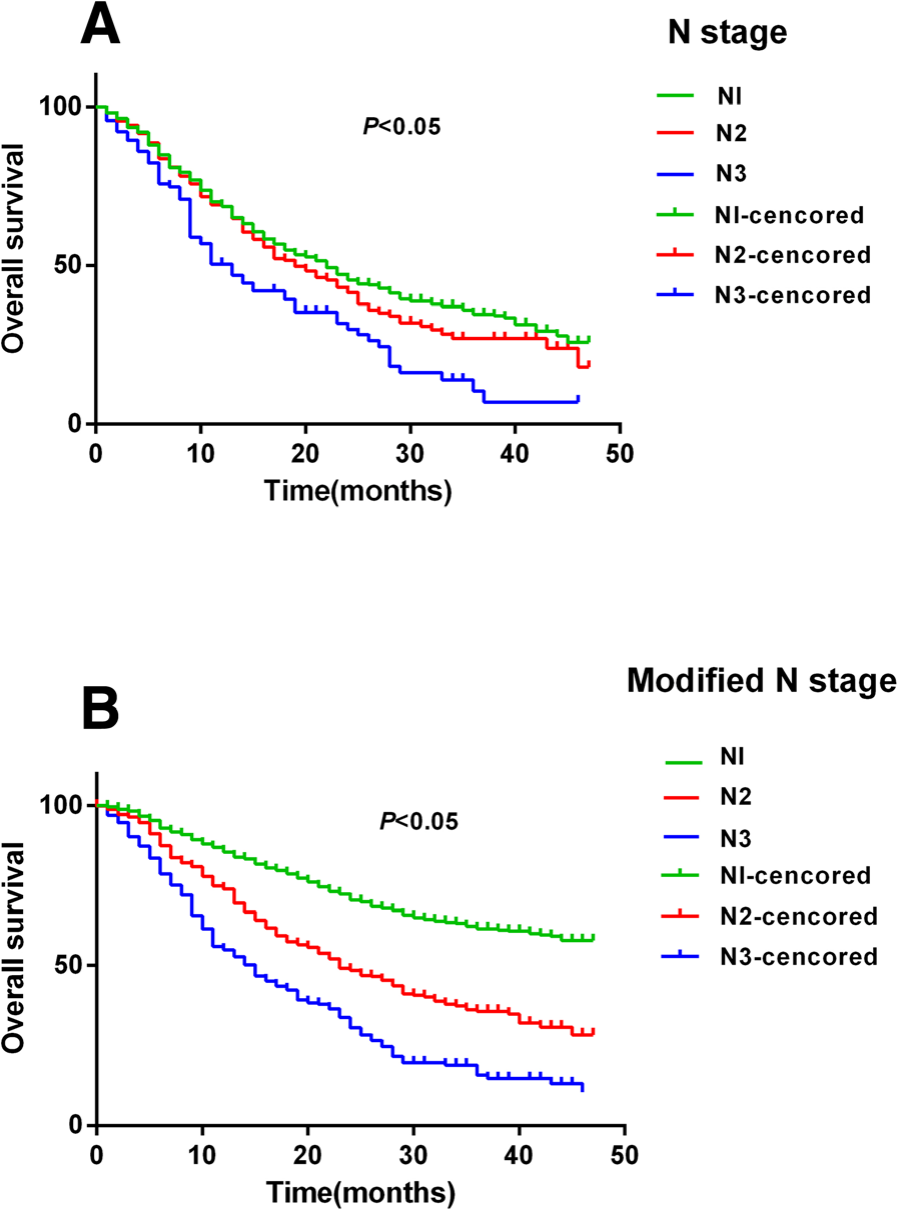
The comparison of survival analysis between the method using N stage of AJCC and the modified N stage, respectively. **a***: The survival analysis on all the patients using the method of N stage coming from AJCC tumor stage system.* **b***: The survival analysis on all the patients using the modified N stage method*

The log-rank test indicated that significant survival differences were confirmed among the N1, N2 and N3 sub-groups of all patients, and the difference of all the patients using the modified N stage method were more significant than AJCC N stage (Fig. 2).

**Fig. 3:**
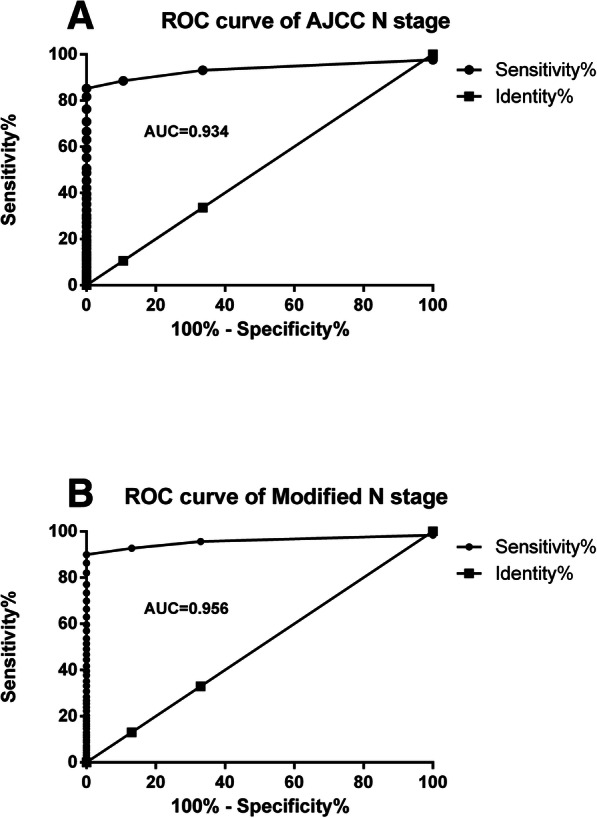
The ROC analysis on the AJCC N stage and modified N stage. *The result of ROC analysis revealed that the area under AJCC N stage curve was 0.934, the area under modified N stage curve was 0.956*

The result of ROC analysis revealed that the area under AJCC N stage curve was 0.934, and the area under modified N stage curve was 0.956, which indicated that the modified N stage could represent the N stage of EC more accurately (Fig. 3).

## Discussion

Because the parameters of the tumor metastasis site may not be stable, patients with EC might be not benefit from the extensive lymphadenectomy [12, 13]. The site would very likely be affected by the extent of operation, whereas _number_PLN collected in lymphadenectomy would not be. Because the PLN was usually enlarged, the surgeon was likely to notice it during lymphadenectomy and would remove it. This means _number_PLN would remain stable regardless of the individual extent of lymphadenectomy and the practice of different surgeons.

Research has indicated that the NLN number could represent the extent of lymphadenectomy in patients with EC, therefore, more NLN removed meant a better prognosis [14]. However, it was controversial how many NLN should be removed in the lymphadenectomy to achieve a better prognosis. Greenstein advised that 18 NLN should be removed in the lymphadenectomy to obtain a better outcome for patients with EC, while 19 NLN was suggested by another study [9, 10]. Baba suggested that 31 NLN should be resected at least in the lymphadenectomy, however, this study found that 31 NLN should be resected only on patients who experienced the three-field dissection for the lymphadenectomy [15]. This finding indicated that _number_NLN was an important factor in the prognosis of patients with EC.

However, the result of the above studies on the advised resected number of NLN was slightly different. The reason for this might be that _number_NLN was easily influenced by the confounders than others. Although _number_NLN could indicate the extent of lymphadenectomy and could reflect the site of tumor metastasis to some extent, a stable cut-off number of NLN removed in lymphadenectomy indicating a better prognosis were not replicated in this study.

The ratio of _number_PLN to _number_NLN or the ratio of _number_PLN to the total number of LN removed in lymphadenectomy could be used to explore the cut-off number which differentiates patients into sub groups with different outcome. This cut-off number could be used for proposing the modified N stage. Dhar et al first reported the ratio of _number_PLN to the total number of LN as a prognostic factor in EC in 2000 [16]. Mariette et al showed that the ratio of _number_PLN to the total number of LN was a strong independent prognosis factor [17]. The above studies demonstrated that the ratio _number_PLN to the total number of LN was as important as _number_PLN regardless of the extent of the lymphadenectomy and the application of neoadjuvant chemoradiation. However, what the best cut-off number was for the ratio of _number_PLN to the total number of LN remained controversial. Several studies proposed 0.2 as the cut-off number for the ratio in their modified N stage regimens, while other studies concluded that the cut-off number for the ratio should be 0.3 [18–21]. In the meantime, Tan suggested that 0.25 might be a more appropriate cut-off number for the ratio than 0.35, which was identified in Shao’s research [22, 23]. The above results indicated that the ratio of _number_PLN to the total number of LN failed to consistently predict the prognosis for patients with EC. The reason might be that none of the above research compared the relative impact of PLN, NLN, and total LN removed in lymphadenectomy on the general prognosis, but they simply used the ratio between them to explore the modified N stage. The criterion of the above-modified N stage would be affected by the research cohort or the proportion of patients.

This study proposed the cut-off ratio of the PLN count to the NLN count based on the results of the Cox proportional hazard model. The procedure in this study was more reasonable than those procedures which directly explored the ratio of the PLN count to the NLN count. Furthermore, the cut-off ratio proposed in this study has been further confirmed using the cross-validation method on cohort data from the SEER database.

The N stage introduced by the 7th AJCC was a regular criterion. The priority of its in predicting prognoses was usually selected to be compared by procedures of modified N stage recently.

A cross-validation study was performed on the modified N stage. The modified N stage was developed from the training population and validated using the validation population, and the survival difference could be replicated in the validation population using the Kaplan-Meier analysis, and the difference of all the patients using the modified N stage method were more significant than AJCC N stage. The survival analysis in this study confirmed that the survival line of subgroups from the modified N stage separated more significantly than that of the N stage of 7th AJCC. Furthermore, the comparison between the modified N stage and the AJCC N stage was performed using the ROC method. The result of the ROC curve analysis demonstrated the superiority of this modified N stage system, which in turn supported the assumption of this study: the relative impact of PLN and NLN on the prognosis based on the results of the Cox proportional hazard model should be considered in the modified N stage.

It was widely accepted that tumor differentiation, _number_PLN and NLN, the tumor stage of 7th AJCC, and organ metastasis were all independent prognostic factors [24, 25]. This study confirmed that finding. Studies also showed that the age of patients was also a prognostic factor [26, 27]. This study confirmed the finding as well. However, based on the analysis result of the Cox proportional hazard model, age only had a small impact on a patient’s prognosis. This result implied that the impact of age on prognosis would only be noticed when using a big cohort. This finding was consistent with our previous research [28].

A recent report revealed that Patients with EC with organ metastasis or distant metastasis in bone had the worst prognosis than others [5]. In this study, patients with tumor metastasis in bone had a worse outcome than those with tumor metastasis in the lungs or liver, but had a better outcome than patients with tumor metastasis in the brain. This finding was consistent with the report which showed that brain metastatic tumors with a primary tumor located in the esophagus only had a mean survival time of six months [29].

Although the current study proposed a reasonable modified N stage for EC, it has several limitations. First, the study was retrospective; its results may be affected by confounding factors that were not controlled for. Second, although the SEER database was prepared according to strict criteria, the data was collected from multiple research centers with different operational habits. As a result, some differences in findings may be due to differences in research center practices. Third, the sample in this study consisted of different pathological types of EC. Because the data did not include which type of EC, this study could not determine whether there was a difference between the application of this modified N stage system or in the ESCC and SCC sub-cohorts.

In summary, based on the results of the Cox proportional hazard model, the study proposed a modified N stage derive from the N stage system of 7th AJCC for EC. The study also identified the reasonability and superiority of the modified N stage using the cross-validation method comp -arising to the N stage system of 7th AJCC. This modified N stage system is a promising step toward more accurately identifying the N stage of EC and in turn, providing more effective treatment for this devastating disease.

## Conclusions

The modified N stage based on the re-adjusted ratio of _number_PLN to _number_NLN can evaluate tumor stage more accurately than the traditional N stage.

## Data Availability

The data sets supporting the results of this article are included within the article.

## Abbreviations

EC: Esophageal cancer
LN: Lymph node
PLN: Positive lymph nodes
NLN: Negative lymph nodes
ESCC: Esophageal squamous cell carcinoma
HR: Hazard ratio
ROC: Receiver Operating Characteristic
LNM: Lymph node metastasis
AJCC: American Joint Committee on Cancer
SD: Standard deviation
IQR: Interquartile range
SEER: Surveillance, Epidemiology, and End Results
JSED: Japanese Society for Esophageal Diseases
